# The role of partners, parents and friends in shaping young women’s reproductive choices in Peri-urban Nairobi: a qualitative study

**DOI:** 10.1186/s12978-023-01581-4

**Published:** 2023-03-09

**Authors:** Anja Zinke-Allmang, Amiya Bhatia, Krittika Gorur, Rahma Hassan, Amy Shipow, Concilia Ogolla, Kees Keizer, Beniamino Cislaghi

**Affiliations:** 1 Department of Global Health and Development, London School of Hygiene and Tropical Medicine, London, UK; 2 Busara Center for Behavioral Economics, Nairobi, Kenya; 3 University of Nairobi, Nairobi, Kenya; 4 University of Groningen, Groningen, Netherlands

**Keywords:** Family planning, Kenya, Gender, Social norms

## Abstract

**Background:**

Contraceptive use among young women in Nairobi remains low despite high general knowledge of family planning (FP) methods. This paper draws on social norms theory to explore the role of key influencers (partners, parents and friends) in women’s FP use and how women anticipate normative reactions or sanctions.

**Methods:**

A qualitative study with 16 women, 10 men and 14 key influencers across 7 peri-urban wards in Nairobi, Kenya. Interviews were conducted during the COVID-19 pandemic in 2020 by phone. A thematic analysis was conducted.

**Results:**

Women identified parents, specifically mothers, aunts, partners, friends and healthcare workers as key influencers on FP. Their interactions with these key influencers varied based on trust, the information they needed about FP, and whether they perceived a key influencer to perpetuate or challenge existing social norms on FP. Mothers were perceived to understand the social risks of using FP and thus could advise on discreet FP use, and aunts were trusted and approachable sources to impartially describe the benefits and drawbacks of FP. Although women identified partners as key FP decision makers, they were cognisant of possible power imbalances affecting a final FP choice.

**Conclusions:**

FP interventions should consider the normative influence key actors have on women’s FP choices. Opportunities to design and deliver network-level interventions which seek to engage with social norms surrounding FP in order to challenge misconceptions and misinformation among key influencers should be explored. Intervention design should consider dynamics of secrecy, trust and emotional closeness that mediate discussions of FP to address changing norms. Further training to change norms held by healthcare providers about why women, in particular unmarried young women, access FP should be provided to reduce barriers for FP access.

**Supplementary Information:**

The online version contains supplementary material available at 10.1186/s12978-023-01581-4.

## Plain language summary

Women’s decisions to use family planning (FP) are influenced by social norms, the unwritten rules of appropriate actions within social networks, and are shaped by advice and information received from key influencers—important individuals who shape what is acceptable within social networks.

The aim of this study was to understand how key influencers uphold and transmit information and norms about FP, and explore who women consult as they make FP decisions and why they consult these key influencers.

We interviewed 16 women, 10 partners and 14 key influencers in peri-urban Nairobi, Kenya.

We found that key influencers for decisions about FP included mothers, aunts, partners, friends and healthcare providers. In making a decision about FP use, women consulted key influencers based on who they deemed trustworthy to keep their FP use secret and described less trust to speak with men about FP in their social networks. Mothers understood the social risks of using FP and so could advise on its discreet use, and aunts were approachable sources of impartial information about FP. Although women identified partners as key decision makers, they were aware that power imbalances might affect final FP choices.

Our findings underscore the importance of FP interventions working with women’s social networks and to address social norms influencing women’s decisions in using FP. Interventions to change FP norms should consider dynamics of secrecy, trust and emotional closeness that affect FP discussions among women’s networks.

**Supplementary Information:**

The online version contains supplementary material available at 10.1186/s12978-023-01581-4.

## Introduction

In 2014, 61.8% of women living in urban areas in Kenya were using modern family planning (FP) methods [1]. This average hides differences in FP access for women living in informal settlements who are disadvantaged in both the availability and quality of FP methods, resulting in lower uptake of FP [2–4] and a higher unmet contraceptive rate than other urban areas [3]. Women’s uptake of FP methods is shaped by global and national level investments in women’s health, access to health services, knowledge and awareness about FP, and gendered power dynamics within household and communities which shape decision-making freedoms [5–7].

A growing number of studies show how social norms—the unwritten rules of common and appropriate actions—within women’s social networks affect women’s decision to use FP methods and the uptake of FP [8–10]. Women do not make FP decisions in isolation, but rather rely on their social networks to verify and gather information on the benefits, side effects, timing, and cost of FP [11]. A study among Kenyan women showed that young women, in particular, value other people’s experiences and advice about FP more than information from health providers when making decisions about FP [12, 13]. Studies in Tanzania [14], South Africa [15] and Angola [16] have also found that social networks strongly influence young women’s decision to use FP. Social norms, and the fear of negative social sanctions, related to the use of FP could deter young women from seeking information or accessing FP [17, 18]. In addition to social norms, Barker and colleagues (2007) link gender norms—social norms that specifically apply to people of a given gender—as among the most important factors that continue to influence FP uptake [19, 20]. Gender norms affecting young women’s use of FP, especially among unmarried young women, include the belief that using FP will be associated with infidelity or prostitution [9, 21] and that women are expected to marry early and have children soon after [22].

Social and gender norms theory draws attention to the importance of ‘key influencers’ who shape and reinforce social norms over time [23–27]. Prior studies show that ‘key influencers’ on young women’s FP decisions, often focusing on family members, partners and friends as key influencers [28, 29]. These key influencers often play different roles in promoting—or stigmatising—the use of FP. Studies show that while parents have been apprehensive about a young woman’s use of FP [30], young women identified their mothers as important and trusted figures to discuss FP choices [28]. Partners are seen as key decision makers in young women’s FP use as they are involved in discussions around desired family size [29, 31, 32]. Young women have also identified healthcare workers as key influencers in their FP decision making since they are trusted to provide accurate information [33]. However since healthcare workers are required to access FP methods, it has also been shown that healthcare workers may restrict method use based on a woman’s marital status, age or parity [34, 35]. Taken together, the evidence suggests that young women access FP information from multiple members of their social circles, despite cultural and normative barriers associated with FP [9, 10, 36]. This paper will explore who the key influencers of FP are within peri-urban Nairobi, with an emphasis on other key actors in social networks than parents and partners, and fills a gap in the literature about how young women navigate their normative context to acquire FP information from various members of their social networks.

In this paper, we draw on qualitative interviews with women and key influencers to examine how partners, parents, aunts, and friends uphold and transmit social normative information about FP, and the implications of these key actors on women’s decision making around FP use. We explore who women consult as they make decisions related to their FP use, why they consulted these key influencers, and how women navigate stigma, support and advice from key influencers, and the varying influence of different individuals within women’s social networks.

## Methods

### Participants and study sites

Phone interviews were conducted with 40 participants across 7 wards in Nairobi in November 2020, during the COVID-19 pandemic. Peri-urban wards were purposively selected based on whether they had health centers and FP services operational at the time of data collection. These wards are part of urban informal settlements in Nairobi with a lack of durable housing, limited access to adequate water, sanitation, refuse collection, and health services.

Participants were 16 women (W) between 18 and 25 years of age, 10 partners (P) and 14 key influencers (KI). Women were randomly sampled from a panel of participants which the Busara Center for Behavioural Economics had recruited between 2014 and 2020. The panel included 66,407 respondents living within Nairobi, 33,829 of which were women. Due to safety considerations for phone-based data collection, women had to have their own (not shared) smartphone to participate in the study. The women interviewed were 20–25 years old (median age = 23 years) and most had some secondary education or higher. Nine women reported being unemployed, two were employed, one was a student, three worked casual jobs and one was a homemaker. More than half of the women interviewed were using contraception.

In the interviews, women first described who they went to for advice on FP. Partners and key influencers were purposively sampled from the panel of participants with similar sociodemographic characteristics to the persons that women described in their interviews. These participants did not reside in the same households and were not from women’s own social networks due to safety and privacy considerations, particularly during COVID-19. Partners were between 23 and 32 years old (median age = 27.5 years), most of whom were employed (7) and had secondary or university education (8). Key influencers were aged 23–52 years (median age = 32 years), most of whom were partnered (11), had 3–4 children (6) and were employed (11). More information about participant sociodemographic characteristics are available in Additional file 1.

### Study design

Qualitative interviews were part of the formative phase of a larger study, which aimed to examine how an online digital media intervention influences social norms around FP use among young women, and served to inform and contextualise the content of that intervention.

The study was a collaboration between researchers from institutions in the Global North (UK, Netherlands) and South (Kenya), who were involved in each stage of the study. We worked together to co-design the study and collaborate on data collection and analysis. We reflected on our positionality, contributions and limitations throughout the study and discussed how our preconceptions and assumptions could shape the study design, analysis and interpretation. To mitigate the bias associated with our positionalities and assumptions, all members of the research team participated in coding, analysis and writing.

The semi-structured interview instrument was designed to explore social norms and sanctions of accessing and using FP, sources for information and decision making on FP, women’s social media use and the effects of COVID-19. To understand young women’s experiences accessing information or FP methods, respondents were asked to reflect on their own experiences and/or the experiences of other women through the use of a vignette of a 23-year-old young woman named Wanjiku who lives in the same neighborhood as participants with her parents and siblings and is considering using modern FP methods including condoms (male or female), pill, patch, ring, diaphragm, injection, IUD, implant and sterilization. Participants were asked: (1) Who are the top two people Wanjiku would consult when deciding about FP that would influence her decision? (2) Why would she reach out to this person(s)? (3) What might this person have said about FP to Wanjiku? (i.e. what opinion/advice or information might they give her about FP, etc.) (4) How might the conversation with this person change what Wanjiku’s will do about FP? More information about the vignette is available in Additional file 2. Accordingly, some answers from participants were from their own perspective (“I”) and from other women generally or the character of the vignette specifically (“she”/“they”/“Wanjiku”). The respondents’ perspectives on who women trust for advice on FP are captured in the findings below.

### Data collection

Data were collected by a team of four trained researchers from Busara who had prior experience conducting qualitative interviews in English and Swahili. Prior to data collection, all researchers participated in a three day training covering topics on social norms, good data collection and management practice, the study’s design and the interview guide. The qualitative instrument was piloted internally, with study staff and the data collectors, and then piloted with 10 participants. The instrument was revised after each pilot. Data collection was phone-based and took place in November 2020 during the COVID-19 pandemic.

Researchers contacted participants who met the inclusion criteria via phone to assess eligibility, explain the study design and schedule a phone interview time. Information about the study was also sent via WhatsApp. Consent was audio recorded, which was iteratively checked-in throughout interviews through: the means of a safe word for participants to use should the conversation no longer be private, check-ins about privacy and comfort, and the use of a vignette to discuss sensitive subjects. Interviews were conducted in either English or Swahili, depending on the participant’s preference. Participants were sent phone credit and a list of local resources and health facilities to access more information on FP and access support for gender-based violence, which were open during the COVID-19 pandemic.

The research team conducted daily debriefs during data collection to discuss emerging themes and to check-in about the safety and comfort of participants during interviews. After data collection, the Busara research manager checked whether consent was sought and reviewed audio recordings. A Busara researcher who had not conducted the interviews translated and transcribed the interviews. The quality of transcription was checked in a random selection of transcripts, which were compared to audio recordings by a study manager who did not conduct or transcribe interviews.

### Data analysis

We conducted a thematic analysis. We developed an initial codebook based on our research questions. To revise the codebook, all authors discussed codes and themes. Following standard procedures in thematic analysis, we first coded the transcript by both using the codes in the codebook, remaining open to emerging new codes. New codes were discussed by all coders as they emerged and were integrated in the codebook. As we coded, we specifically paid attention to potential deviances from what most participants said, mindful of the importance of analysing the data both with and against the grain [37]. As we continued this first step of the analysis, we reached a point of saturation, when the raw data did not seem to yield any codes. Then we gathered similar codes in larger themes. For the current paper, we focused on themes that described (1) who women would consult as they make decisions related to their FP use and (2) how and why they consulted these key influencers.

### Ethical approval

Permission to conduct this study was obtained from Strathmore University, Nairobi (ref SU-IERC0898/20) and the London School of Hygiene and Tropical Medicine (ref 22480). Participants provided verbal consent prior to interviews and identifying information was removed from transcripts prior to analysis.

## Results

Participants identified five groups of people who influenced their decision making about FP: (1) Parents, (2) aunts, (3) partners, (4) friends and (5) healthcare workers. While most women, partners and their key influencers said that parents were important to consult on matters related to FP, nearly every woman said that mothers were more supportive than fathers. Specifically, mothers could understand the social risks of using FP and could advise their daughters on how to do so in discreet ways. Besides mothers, aunts were seen as approachable sources of information on available FP methods including the benefits and drawbacks of using them. Participants saw women’s partners as key decision makers and friends as important sources of emotional support. Lastly, women and their key influencers trusted healthcare workers to be impartial, confidential, and knowledgeable sources of information and advice on which FP methods to use. In passing, two women identified other trusted community members, including religious leaders and a mother’s older friend, as sources of FP information. We discuss the role of each of these key influencers in shaping how women engage with FP information, services, and methods.

### Parents

Women largely preferred mothers to fathers when they needed to talk about FP. In explaining their choice, most women echoed one participant who said: *“A mother wants what’s best for her [daughter]” (W_12).* Partners and KIs agreed, suggesting mothers have personal experiences to advise their daughters: *“They can relate with each other—there are those daughters who are free with their mothers and can share anything with them” (P_21).* This emotional closeness was evident when some women described that mother’s advice-giving centered around experiences of having “suffered” from financial or health consequences of having many children or having children early. One woman said that a mother would tell her daughter to learn from her experience and use FP because *“the mum has gone through a tough life, bearing a lot of children because she never wanted to use family planning” (W_8).* Several women described that “suffering” would motivate mothers to recommend FP so their daughter could avoid the constraints associated with childbearing: *“The mum will advise her to use family planning not to end up like her” (W_8).* Other women noted that if a mother is not aware that she had “suffered” in the past, then she will not recommend FP as she would not understand the value of FP:

*“It will depend on the background of Wanjiku’s mother. If she grew up in a good family, she will advise her well [to use FP]. You see, in our local area, we follow our parents’ footsteps and what they do. If you see that she is a sufferer who doesn’t even understand herself, what kind of advice will she even give me.” (W_4)*

Despite relatively high trust in their mothers, several women expressed reservations about fully confiding in their mothers. One woman, in response to the vignette where a daughter considers speaking with her mother on FP, said*”I don't know if she trusts the mom. I won’t say she trusts the mom yet but she just wants to make her aware of it [her family planning use]” (W_14).* One reason for hesitating to share openly about FP use was the worry about tarnishing the family’s or their own reputation. A few women acknowledged that their actions of accessing or using FP could still confer shame to their families*: “Her parents will take her to be immoral”* (*KI_37*). Mothers, likewise, were susceptible to community judgment in relation to an unplanned pregnancy in addition to FP: *“Her mother can notice she has a boyfriend and she can advise her to use a condom with him and not bring shame to her” (W_12).* A few women added that being seen purchasing FP could also be shameful and anticipated a mother would advise being more secretive, *“[The mother] will be like, did you have to go there [to the chemist] while everyone is seeing you? You are embarrassing me*” (*W_1*). Women recognized that mother’s reputations were closely tied to their own reputations, however, this was sometimes in tension with wanting a better life for their daughters: *“Her mom will see that instead of Wanjiku bringing her a grandchild in the house, it is better she protects herself [by using FP]” (W_4).* Women weighed prescribed norms of chastity before marriage, the fear of disappointing their families by using FP to prevent shame or pregnancy alongside the risk of ‘suffering’ associated with early pregnancy.

While participants overwhelmingly referring to mothers as the key parent women would speak to, almost none of them held fathers in the same regard. Only one participant said fathers would be supportive of their daughters discussing or using FP: *“Because they [fathers] can’t let their child down with bad advice” (KI_31).* Participants said women wouldn’t talk to their fathers primarily for two reasons: They wouldn’t know how to start a conversation on such a sensitive topic with them, and they would be scared of their fathers' reaction. While mothers were considered a “*fellow woman*” (*W_4*), a daughter “*will not even have an idea how to start such a conversation with the father*” (*W_8*). Most participants said women would be ashamed to talk about FP with their fathers: *“It [talking about FP use] is shameful, sharing that with the father” (W_8).*

Not only were participants ashamed to talk to their fathers about their reproductive health, but a few also mentioned fearing fathers’ possible reactions. One woman, for instance, said, *“her father might be interested in knowing where Wanjiku learnt about family planning and even become a nuisance to Wanjiku’s mother”* (*W_9*). Partners and KIs said that fathers would react negatively to learning about a daughter using FP, as that would imply she was having premarital or early sexual intercourse, *“especially when they realize their children have started involving in many things [sexual activities]”* (*P_24*). In some circumstances the anticipated reaction from fathers could be more severe than in others. A couple participants noted that a father’s anger about their daughter’s FP use could sometimes escalate to violence: *“[The father] can beat her up”* (*W_15*). Partners also suggested that there was potential violence if a father finds out about his daughter accessing or using FP: *“Because if I were the Dad and she happens to come for such advice [about FP], I would beat her up. There are issues that are supposed to be shared with the mum and not the dad”* (*P_21*). A few participants described a risk to women who live with their parents being forced to leave the home due to parents learning about their FP use: *“She is afraid that this information will get to her parents who will chase her from home”* (*P_19*). Although only a few participants noted strong sanctions against speaking about FP to fathers, most women anticipated negative reactions and described avoiding the subject or their fathers finding out about their FP use.

### Aunts

Aunts were considered trusted confidants for a variety of personal matters for women, including FP use. Participants said women would be more comfortable approaching aunts than their parents, as aunts would be more likely to speak openly: “*The aunt may understand her and talk to her about the benefits and disadvantages of using family planning. The aunt will advise her right compared to her sister or mother” (KI_35).* Participants thought that an aunt would share both the benefits and limitations of FP openly and would ultimately recommend using a contraceptive method: “*The aunt might have given her the good and the bad side of the family planning and tell her to use it because getting a kid during this time is really hard—there is no money” (W_6).* Similar to mothers, aunts’ recommendations were anticipated to be based on past personal experiences, such as being pressured into early sex or being made false promises by boyfriends:

*“The aunt will tell her that ‘you may use family planning but if the boyfriend doesn’t want you to use it, then he is not safe for you because a person who loves you will not want you to have a family at an early age especially when you are not financially stable’. She will give a lot of advice because Wanjiku approached her knowing she is a good person and will set an example to her.”* (*W_6*)

Aunts’ impartiality was deemed valuable in learning benefits and limitations to FP when women make their own decisions about using FP.

Similar to other adults in the family, comfort with approaching aunts was connected with how close women were to their aunts, and perceived judgement: *“It is not easy to approach a grown up and open up because questions will rise up and she might be afraid of the aunt asking so many of them like how many friends she has or where she goes for parties” (W_6).* One woman said it isn’t uncommon to use a cover story of a friend wanting information on FP to approach aunts about FP rather than asking directly about their own circumstances. Despite anticipating being asked many personal questions in the pursuit of knowledge or advice about FP from an aunt, this participant noted that women might still go to their aunts for advice because “*[the aunt] has a good family and [a woman] would want to be like her” (W_6).*

### Partners

Although consulting family members on FP provided women advice from trusted sources, nearly all women described partners as key decision makers. Many women also felt partners should understand each other which was connected to the expectations of healthy relationships:

*“Relationships are all about understanding each other. If the boyfriend loves her, he should then be able to understand Wanjiku [wanting to use FP]. That doesn’t mean she has to do stuff and not tell the boyfriend—he should be understanding.” (W_9)*

Several women anticipated that their partner would oppose a decision to use FP for various reasons. One woman described the general imbalance in decision making power in relationships with men when it came to using FP: *“Some husbands think they are the only ones who can make decisions in the home. A woman has no right to talk” (W_12).* One man linked this imbalance specifically to FP where “*[a woman using FP] will depend on what the boyfriend wants at that particular time*” (*P_23*). Some women anticipated opposition from partners about using specific FP methods and suggested that a woman *“should ask her boyfriend to use a condom, but if he doesn’t agree, she should use pills to avoid having children at an early age”* (*W_6*). Other women described using discreet modern methods, such as the pill, but claim to use traditional methods when asked about their method use: *“In such a case where women are not allowed to use family planning, a wise woman will use it and then say she is using the safe-day method when confronted”* (*W_9*). Partners and other KIs agreed that a woman will likely use FP in secret if her partner does not agree to use a visible method.

Many women, partners and KIs described a partner’s concerns about the possible side effects of FP, in particular about FP’s potential impact on a woman’s future fertility where one KI said, “*if [a partner] finds out that it is Wanjiku’s fault [for not becoming pregnant because she took] family planning for a long time, she may lose her marriage*” (*KI_35*). A partner linked the concern about future fertility to societal values where “*in the African culture, people are termed to be wealthy according to the size of the family they have; the larger the family, the wealthier they consider you*” (*P_21*). Participants also described partner’s concern about FP’s side effects on the woman’s body where one woman said *“[partners] will not support [using FP] and they say family planning tampers with a woman’s body” (W_7).* Similarly, a partner remarked:

*“Men tend to think that family planning changes the woman’s body and that reduces the man’s sexual desires. There is a way in which it affects the man. They say the woman feels cold during intercourse and that affects the man sexually.”* (*P_23*)

Women described that using FP could indicate a lack of trust in the relationship or might be perceived by partners as a license to have sex with multiple men. One woman said, “*so, when [women] use family planning, they [partners] see a woman who is a whore, or who sleeps with many men and doesn't want to be caught”* (*W_14*). This perception creates barriers for women to negotiate FP use transparently with their partners, because “*even telling [a partner] to use one [condom] might result in them asking if the women think they are being unfaithful and this might result in arguments*” (*W_6*). Thus, women often described partners approving of FP use during marriage as a means of birth spacing, rather than a safeguard during premarital sex. The risk was even greater if women failed to share their usage or chose not to consult their partner, to the point of being outed to community elders as deviants or being positioned as unfaithful:

*“Some men end up reporting their wives to the church pastors if they find out they are using family planning against their wish then the pastor calls the wives and questions her as to why she is using family planning and why she didn’t tell the husband about it.” (W_5)*

While partners were identified as very important to talk to in the final decision making on FP use, many women also suggested speaking to other people in their social network about what methods to consider, such as friends, their mothers or a healthcare worker. Where families provided advice to avoid shame in FP use and partners were important decision makers in FP, close friends were important in hearing others’ experiences with various FP methods.

### Friends

While women’s partners were often involved in the decision on FP use, most women also sought out emotional support and perspectives from their close friends. Friends were seen as being in similar situations, sharing and discussing personal experience in using FP methods were valued: *“She is free with her [friend], they are of the same age and they can reason together. Secondly, she is someone she can lean on and maybe her friend has told her about family planning” (W_7).* Women described their friends as close confidants with whom they would be comfortable talking about FP with, *“you know, a girl has two friends whom she shares everything with” (W_14)*. When women established trust with their friend, women felt that friends could speak to the advantages and obstacles of both obtaining and using FP. Although most participants suggested that friends are trusted sources to confide in about FP use, one woman recognized that friends might gossip and anticipated being vulnerable to judgments of promiscuity: *“Wanjiku should avoid telling friends whom she doesn’t trust because they will gossip about her with other people” (W_9).* Conversations with friends about FP were ongoing and often encapsulated the multiple choices women had to make regarding if, when and how to use FP:

*“So this is what she [friend] would say: ‘Why do you want to use it? It’s a good idea but you should know that each and every method has its own consequences. Because the pill was not so good for you. You told me before. I will tell you to try the injection. If it's not good, try the lower period of time [3 month injection]. If it's not good, try another method or go for advice from a doctor and try another good method.’” (W_14)*

Participants were divided on whether friends would recommend FP to young women. This division was largely based on the fear that women might change their mind and want to have children in the near future while still using long-term methods: *“[A young woman] might decide to use an injection that lasts for a year then come across a serious man who wants to have a child immediately and Wanjiku fails to have them because of the 1 year injection she has on her. That’s why [a friend] will suggest the one for three months” (P_23).* Due to this concern about future fertility, a couple of women said that friends might advise having a child before using FP: *“[A friend] might ask her why she wants to use family planning—she doesn’t have children and that she ought to have waited” (W_5).* One key influencer similarly suggested that a friend would tell a woman that, “*she has to get a child first and after the child, she can decide to use family planning*” (*KI_27*). While our findings are divided on whether these friends would recommend FP use, most women said their friends would refer women to visit a doctor for accurate and tailored information on which FP methods to use.

### Healthcare providers

Whereas women sought emotional support from parents, partners and friends, they turned to healthcare providers (HCPs) as credible and confidential sources for information on FP. All participants expressed how doctors are largely a trusted source of information since “*they have the best advice for her that she can’t doubt”* (*KI_32*). One woman similarly said, *“She trusts the doctor because I know that once you get to the doctor, the conversation and information is confidential” (W_4).* Another woman said that trusting a doctor was also based in the hospital or clinic setting which would preserve their privacy: *“When you are in a hospital no one knows you or knows where you come from, you are always comfortable to share any information or seek any information because you know that there is no way that person will connect to where you come from or meet anybody you know” (W_4).* This sense of privacy was the main reason doctors were identified as a trusted source of information.

A few participants anticipated being questioned or judged by HCPs about using FP on the basis of their age or marital status. Adolescents faced doctor’s bias about early sex which would increase barriers to accessing FP: *“With injections, the doctor may ask why a small child is going for family planning. You do know how the society we live in is” (W_4).* These biases extend to the number of children they have: *“The doctor needs to ask ‘why do you want to use the method. And how old are you? Do you have a child? And are you sure—hundred percent—you need to start using family planning methods?’” (W_14).* A few women anticipated that women who are not married or who do not yet have children would be recommended short-term non-hormonal methods: *“I think the doctor would advise her to use different drugs if she is married and others if she is not married. If she has children she will be given different drugs and if she is yet to have children she will be advised to use condoms” (W_12).* One woman also suggested that HCPs could hold biases that women might want to access FP to cheat on their husbands, *“[The doctor] might have advised her to sexually stick to one man after giving her the family planning” (W_9).* What this woman said echoed what others mentioned about their partners concerned with their possible infidelity, which, in turn, is suggestive of a larger system of norms connecting use of FP and infidelity that expands beyond the couple. While most women identified doctors or other healthcare workers as supports in accessing FP, a few had reservations about whether healthcare workers might act as gatekeepers and pose as potential barriers to accessing FP.

## Discussion

We drew on 40 interviews with women and their key influencers in peri-urban Nairobi. Women reported seeking support and information with a variety of key influencers largely including mothers, partners, aunts, friends and HCPs, and discussed how decision making about FP was sometimes shared with these key influencers and was sometimes hidden. Other studies have underscored how women’s social networks are the primary source of information on FP in Nairobi, where key influencers’ perceptions about FP heavily influence the FP decisions women make [9, 13–16, 18, 28], and shape whether women can access the resources (money, transport, time) to visit clinics or pharmacies to seek FP [38–41]. Elsewhere, we show how COVID-19 further entrenched the reliance on key influencers. Our findings build on this literature on how social networks affect women’s health and to uncover how women navigate norms around FP within their social networks to gather information to make a decision on using FP.

Trust and secrecy were central for women in deciding who to speak with about using FP. Although women sought information from multiple sources before making a decision about FP use, they chose to consult those they trusted most to confide their FP use to within their social network. Trust was critical, as women wanted to keep their FP use secret to avoid social sanctions from, for instance, their neighbours or fathers. Women described trusting close female friends and relatives more than men in their social circles: Mothers were a particularly important source of information on how to maintain the secrecy of their FP use. Trust and comfort in discussing sexual health topics between mothers with their daughters is a common theme in other studies [30, 42, 43]. Our findings are in line with Wamoyi and colleagues (2010) who linked trust in speaking to mothers about sexual health matters to a mother’s ability to offer advice based on their own personal sexual or reproductive health experiences [44]. Similar to our findings, other studies have also described fathers as the least accessible parent for women, describing father’s negative reactions to discussing FP as a barrier for women in both seeking information about FP and using FP [30, 44]. We found a few young women anticipated a father’s reaction would be violent if FP was discussed, underscoring the uncertainty that women can face in finding supporters of FP use in their social networks. The acceptability of speaking to mothers but not fathers about FP reflects gendered norms about who it is appropriate to speak with about sexual health by young women [9, 41, 44]. To navigate the possibility of negative reactions with other family members, and where women are uncertain about the trustworthiness of a key influencer (e.g., aunts), they might begin by describing the situation of a friend before disclosing their own FP use. This is particularly salient for single women who face additional stigma from using FP as compared to partnered women, who experience other forms of stigma and barriers to contraception use [45–48].

We found that some partners opposed FP as it is associated with infidelity, as seen in other studies [9, 32, 49]. While other research has found that men, irrespective of their knowledge on FP, want to be involved in the decision of FP within relationships [49, 50], our findings provide further evidence that women anticipate partners to oppose FP use, while also believing that women should talk to their partners about their FP use. In anticipating opposition from partners, some participants suggested that women should use FP irrespective if their partner disagreed. Women advising others to use FP regardless of their partner’s opposition, suggests a shift in norms within peri-urban wards in Nairobi that prioritizes a women’s FP decision over a partner’s preference as FP becomes more widely used [1, 51]. However, participants also discussed the burden of secrecy, including the fear that partners or key influencers might tell others in the community about their FP use.

Many women identified HCPs as key sources of knowledge and information about FP, describing HCPs as the most confidential option for accessing reliable, accurate information about FP. However, young women in Sub-Saharan Africa have limited youth-friendly FP services which increase barriers to FP access [16, 52], where existing barriers have been exasperated and additional barriers were reported during the COVID-19 pandemic [36]. HCPs acting as gatekeepers to FP access has been documented for both unmarried and married women, where accessing FP can be restricted by HCPs due to woman’s age or marital status as a result of HCPs being influenced by the norms within the community they serve [16, 34, 35, 53]. Women share their experiences with HCPs within their social networks to warn other women navigating the normative context around FP, resulting in women not solely relying on HCP, but rather accessing or confirming information about FP from their friends, who are trusted sources [9, 49]. While women also describe the support they anticipate from friends as mixed, they highlight the importance of sharing anecdotal information or experiences with their friends to make informed decisions about FP. Navigating various sources of information and avoiding shame—both for women and their families—underscored the important role of friends and healthcare workers to provide judgement-free, accurate information. While accessing information about FP from key influencers is important to women, they are thus exposed to the norms and misconceptions perpetuated in their normative context about FP before they make their final decision on using FP.

This study has several limitations. While our findings provide insights into the people women trust and discuss FP with, as well the fears and concerns women have about these conversations, the findings in this paper cannot be generalized and do not represent the experiences of all young women living in these wards in Nairobi. The sample size of this study was not large enough to explore differences by age, marital status or other demographic characteristics. All interviews were conducted by phone due to the COVID-19 pandemic, which may have affected the information participants felt comfortable sharing. However, especially during the COVID-19 setting, phone based interviews were advantageous to prevent the spread of the virus and evidence has found that participants might find this mode more convenient to participate in the study [54]. Nonetheless, this study fills a gap in the literature on how young women in peri-urban areas in Nairobi have navigated their social networks and normative context to access information on FP which influences their health and FP decision making.

Our findings have several implications for policy and practice on FP in Kenya. First, the role of key influencers should be central in the development of interventions to improve the uptake of FP or change social norms about FP use. The role of key influencers is ongoing, requires trust, many conversations, and different levels of secrecy and disclosure about FP. FP interventions that include women’s social networks should focus on the relational dynamics between women and their key influencers, by considering the dynamics of secrecy, trust, and emotional closeness that mediate who women talk to about FP in the context of changing norms and addressing the barriers to FP. Such efforts should not be one-off, or only focused on education or awareness raising activities with key influencers, but engage with the relational context and ongoing nature of these conversations. Secondly, our findings about the social norms held by HCP suggests the need for further training to change norms that HCP hold, in particular about why unmarried women in particular access FP. Thirdly, involving men in FP interventions should include efforts to address harmful norms and attitudes about FP. Partners and parents are key influencers in women’s decision to use FP, where negative norms and attitudes towards FP might result in harm to women and low uptake of FP. Finally, future research could explore which relational spaces are most effective to transmit accurate information to women deciding on whether or not to use FP and further explore the norms held by key influencers. In particular, longitudinal qualitative studies would be instructive in understanding the ongoing conversations women have with key influencers in their social networks and how they navigate questions of shame and secrecy as they make decisions about FP.

## Conclusion

Drawing on data from 16 young women, 10 partners and 14 key influencers in Nairobi, we found that women drew on their social networks as primary sources of information on FP therein navigating their normative context to find support from mothers, partners, aunts, friends and healthcare workers. This study shows how feelings of trust, a desire of secrecy, and a need for FP information shaped how women chose to speak to multiple people in their social networks based on the type of support they are seeking for their decision on using FP. We find that the process of navigating social circles for trusted people to discuss FP with is an ongoing process, where women seek people who will keep their FP use secret to avoid shame and sanctions. These findings highlight the importance of FP programmes to connect with women’s social networks to engage key influencers and to share accurate FP information through women’s social networks. Such efforts could be important in reducing shame and stigma associated with FP and improving access to information about FP.

## Supplementary Information

**Additional file 1.** Participant characteristics.

**Additional file 2. **Vignette Interview Tool.

## Data Availability

The datasets generated and analyzed for this study are not currently publicly available of the sensitive nature of the qualitative data that can be linked back to individuals. They are available from the author on reasonable request.

## Abbreviations

COVID-19: Severe acute respiratory syndrome coronavirus 2, also known as SARS-CoV-2
FP: Family planning
HCPs: Healthcare providers
