# Supplementary material for: The role of partners, parents and friends in shaping young women’s reproductive choices in Peri-urban Nairobi: a qualitative study
Source: Reprod Health. 2023 Mar 9;20:41. doi: 10.1186/s12978-023-01581-4 (PMC9997433; doi:10.1186/s12978-023-01581-4)
Supplement: Supplementary file 1 — Additional file 1. Participant characteristics. [file 12978_2023_1581_MOESM1_ESM.docx]

**Additional File 1. Participant Characteristics**

| **Characteristics** | **Women (n=16)** | **Partners**  **(n=10)** | **KIs**  **(n=14)** | **Total**  **(n=40)** |
| --- | --- | --- | --- | --- |
|  | **n** | **n** | **n** | **n** |
| **Age** | | | | |
| 18-25 | 16 | 4 | 3 | 23 |
| 26-30 | N/A | 5 | 4 | 9 |
| 31-52 | N/A | 1 | 7 | 8 |
| **Sex** | | | | |
| Female | 16 | 0 | 10 | 26 |
| Male | N/A | 10 | 4 | 14 |
| **Education** | | | | |
| Primary or less | 2 | 2 | 4 | 8 |
| Secondary | 11 | 5 | 7 | 23 |
| University or higher | 3 | 3 | 3 | 9 |
| **Marital Status** | | | | |
| Single | 8 | N/A | 3 | 11 |
| Married or partnered, living together | 8 | 7 | 10 | 25 |
| Married or partnered, living apart | 0 | 3 | 1 | 4 |
| **Contraceptive Use** | | | | |
| Not using contraceptives | 6 | 3 | 4 | 13 |
| Using contraceptives | 10 | 7 | 10 | 27 |
| **Number of Children** | | | | |
| 0 | 7 | 4 | 1 | 12 |
| 1-2 | 9 | 6 | 7 | 22 |
| 3-4 | 0 | 0 | 6 | 6 |
| **Employment** | | | | |
| Employed | 2 | 3 | 6 | 11 |
| Casual employment | 3 | 4 | 5 | 12 |
| Unemployed | 9 | 3 | 3 | 15 |
| Other (student, homemaker) | 2 | 0 | 0 | 2 |
