# Supplementary material for: The role of partners, parents and friends in shaping young women’s reproductive choices in Peri-urban Nairobi: a qualitative study
Source: Reprod Health. 2023 Mar 9;20:41. doi: 10.1186/s12978-023-01581-4 (PMC9997433; doi:10.1186/s12978-023-01581-4)
Supplement: Supplementary file 2 — Additional file 2. Vignette Interview Tool. [file 12978_2023_1581_MOESM2_ESM.docx]

**Additional File 2. Vignette Interview Tool**

| **Vignette story** | **Questions posed after section of story** |
| --- | --- |
| I would like to tell you a story about a young woman called Wanjiku. The story is fictional, but I would like you to pretend that Wanjiku is a woman who lives in the same neighbourhood as you. She is 23 years old and lives with her mother, father, and siblings. One day, Wanjiku sees an advert about modern family planning methods and starts to think about using them. Can you picture Wanjiku? | 1. Why do you think Wanjiku might want to start using a family planning method?  2. Why do you think Wanjiku could decide not to begin using family planning? |
| Let’s continue the story about Wanjiku. She decides to ask a friend about this decision. The next day she meets a very good friend of hers, Grace. Grace is the same age - also 23 years old - and comes from the same neighbourhood as Wanjiku. Wanjiku tells Grace that she is considering using family planning and asks Grace for advice. | 3. What do you think Grace will advise her to do?  4. When does Grace think is the right time for Wanjiku to start using family planning methods?  5. Remember Wanjiku’s friend Grace is also 23 years old. What would Grace suggest as possible family planning choices? Why would she suggest them?  6. Now let’s think about women in the community. What are situations in which people might think it’s NOT okay for a woman to use modern family planning methods?  7. Are there any situations in which people think it IS okay for a woman to use modern family planning methods? Explain.  Prompts included asking about age, marital status, having a boyfriend, the number of children a woman has, religion, or other factors. |
| To continue the story, after Grace has spoken with Wanjiku, Wanjiku decides that she wants to use a family planning method. She tells Grace that she also wants to talk to other people about this decision. Now I want you to think about all the other people Wanjiku could talk to. | All participants  8. Who are the top two people Wanjiku would consult when deciding about family planning that would influence her decision? |
|  | Questions for Women  For each person (2) the following questions were asked:  9. Why would she reach out to this person? Probe: Why does she trust this person? When Wanjiku reaches out to [PERSON] to talk about family planning, how would she contact them? (in-person, phone, whatsapp, facebook etc.)?  10. What might [PERSON] have said about family planning to Wanjiku? (i.e. what opinion/advice or information might they give her about family planning, etc) Probe: Did this person have a positive/negative response to talking about family planning? How would this make Wanjiku feel? What is one piece of advice this person may have given? What is an experience that [PERSON] might share with Wanjiku?  11. How might the conversation with [PERSON] change what Wanjiku’s will do about family planning? Probe: Is there anything Wanjiku would not feel comfortable asking this person? Does Wanjiku feel she still needs to find other information after speaking with this person to make a decision? Who else would [PERSON] tell Wanjiku to talk to about family planning? |
|  | Questions for Partners & Key Influencers  9. Why are these people important to her?  10. What might they have said to Wanjiku?  11. How would Wanjiku contact these people? |
| I’d like to finish the story about Wanjiku. After she speaks with the people she trusts, Wanjiku decides to visit a chemist to ask for some information about her choices. However, her mother’s friend overhears her asking for information on family planning methods. | 12. How does Wanjiku feel about her mother’s friend seeing and overhearing her questions to the chemist?  Probe: What consequences would Wanjiku be afraid of if her mom’s friend knew that Wanjiku is using family planning? Is she worried about her family learning about her visit to the chemist? Who else is she worried about knowing she had visited the chemist for family planning information? What would her family’s reaction be to Wanjiku starting family planning? Who should Wanjiku absolutely avoid telling about her decision to use family planning? |
| *Note that data collectors were trained to provide further probes based on their experience and the interview.* | |
